# Transcriptome and Proteome Analysis in LUHMES Cells Overexpressing Alpha-Synuclein

**DOI:** 10.3389/fneur.2022.787059

**Published:** 2022-04-11

**Authors:** Matthias Höllerhage, Markus Stepath, Michael Kohl, Kathy Pfeiffer, Oscar Wing ho Chua, Linghan Duan, Franziska Hopfner, Martin Eisenacher, Katrin Marcus, Günter U. Höglinger

**Affiliations:** ^1^Department of Neurology, Hannover Medical School, Hanover, Germany; ^2^Medical Faculty, Medizinisches Proteom-Center, Ruhr University Bochum, Bochum, Germany; ^3^Medical Proteome Analysis, Center for Proteindiagnostics (PRODI), Ruhr University Bochum, Bochum, Germany

**Keywords:** Parkinson's disease, alpha-synuclein, transcriptome, proteome, vesicular transport, synapse, lysosome

## Abstract

LUHMES cells share many characteristics with human dopaminergic neurons in the substantia nigra, the cells, the demise of which is responsible for the motor symptoms in Parkinson's disease (PD). LUHMES cells can, therefore, be used *bona fide* as a model to study pathophysiological processes involved in PD. Previously, we showed that LUHMES cells degenerate after 6 days upon overexpression of wild-type alpha-synuclein. In the present study, we performed a transcriptome and proteome expression analysis in alpha-synuclein-overexpressing cells and GFP-expressing control cells in order to identify genes and proteins that are differentially regulated upon overexpression of alpha-synuclein. The analysis was performed 4 days after the initiation of alpha-synuclein or GFP overexpression, before the cells died, in order to identify processes that preceded cell death. After adjustments for multiple testing, we found 765 genes being differentially regulated (439 upregulated, 326 downregulated) and 122 proteins being differentially expressed (75 upregulated, 47 downregulated). In total, 21 genes and corresponding proteins were significantly differentially regulated in the same direction in both datasets, of these 13 were upregulated and 8 were downregulated. In total, 13 genes and 9 proteins were differentially regulated in our cell model, which had been previously associated with PD in recent genome-wide association studies (GWAS). In the gene ontology (GO) analysis of all upregulated genes, the top terms were “regulation of cell death,” “positive regulation of programmed cell death,” and “regulation of apoptotic signaling pathway,” showing a regulation of cell death-associated genes and proteins already 2 days before the cells started to die. In the GO analysis of the regulated proteins, among the strongest enriched GO terms were “vesicle,” “synapse,” and “lysosome.” In total, 33 differentially regulated proteins were associated with synapses, and 12 differentially regulated proteins were associated with the “lysosome”, suggesting that these intracellular mechanisms, which had been previously associated with PD, also play an important role in our cell model.

## Introduction

Parkinson's disease (PD) is the second most common neurodegenerative disorder after Alzheimer's disease. The pathophysiological mechanisms involved in neurodegenerative disorders such as PD are not yet fully elucidated. The first locations in the brain that are affected by PD pathology are the olfactory bulb and nuclei of the glossopharyngeal and vagal nerve in the brain stem. Eventually, the pathology reaches the dopaminergic neurons in the substantia nigra pars compacta in the midbrain (1). The demise of these dopaminergic cells is responsible for the typical motor symptoms of PD, including bradykinesia, rigidity, and tremor at rest (2). However, also non-motor symptoms, including depression, constipation, loss of the sense of smell, sleep disturbances, and later-stage cognitive impairment, can occur (3). Histopathologically, PD is characterized by the presence of intracellular proteinaceous inclusions in neuronal cells termed Lewy bodies (LBs). These mainly consist of aggregates of a small protein called alpha-synuclein (αSyn) (4, 5). Thus, PD is considered to be a synucleinopathy. Very rare point mutations in *SNCA*, the gene-encoding αSyn, as well as *SNCA* duplications and triplications, lead to autosomal dominantly inherited Parkinson's syndromes, whereas the severity of the disease increases with the *SNCA* copy numbers (6–8). Furthermore, multiple genome-wide association studies showed that single nucleotide polymorphisms in *SNCA* are risk factors to develop sporadic PD (9, 10). This underlines the importance of αSyn in the pathophysiology of PD. Nevertheless, the physiological function or functions of αSyn are not yet fully understood. It is believed that αSyn plays a role in synaptic transmission and plasticity (11). Furthermore, it is not yet fully understood which αSyn species lead to cytotoxicity. Whereas, αSyn is considered to be a monomeric protein in its natural form, LBs contain large aggregates. It is generally believed that some forms of oligomeric or fibrillary αSyn that are generated in the aggregation process from monomers to LBs are toxic (12). Contemporary medical treatment of PD is purely symptomatic and is mainly based on the substitution of the dopaminergic deficit or the blockage of dopamine degradation. So far, there is no therapy available with a proven effect on the progression of the disease, not to mention a complete halt or even reversal of the pathological process (13, 14). For better understanding of the pathophysiology of PD and αSyn in particular, cell and animal models are used. LUHMES cells are derived from dopaminergic neurons of 8-week-old human embryos, the cells, the demise of which causes PD motor symptoms in adult patients. The cells have been immortalized by cloning in the myelocytomatosis viral oncogene (v-myc) under a tetracycline-controlled transcriptional activation (Tet-Off). The Tet-Off system enables the proliferation of the cells into large amount. However, after addition of tetracycline and different growth factors, proliferation stops, and the cells can be differentiated to postmitotic dopaminergic neurons that resemble the dopaminergic cells of the substantia nigra (15), with expression of neuronal and dopaminergic markers in particular. We previously presented a model, in which overexpression of human wild-type αSyn in LUHMES cells using adenoviral vectors leads to approximately 50% cell death, 6 days after transduction (16). This model can be used to investigate potentially neuroprotective drugs in a high throughput setting, and results from the cell model (i.e., protection from αSyn-induced toxicity) can be reproduced in PD mouse models (17), supporting the relevance of findings in the LUHMES cell model. In this model, obvious cell death occurs 6 days after induction of αSyn overexpression, whereas, 4 days after transduction, no or only very little cytotoxicity can be observed (16). In the present study, we performed an analysis of the transcriptome and proteome of LUHMES cells overexpressing αSyn in comparison to LUHMES cells expressing GFP as control protein to identify changes in the differential regulation of the expression of genes and proteins as a consequence of αSyn overexpression. Since αSyn overexpression led to marked toxicity in LUHMES cells between Days 4 and 6 after transduction, we analyzed cells at Day 4 after transduction before marked cell death appeared.

## Materials and Methods

### LUHMES Cell Culture

For proliferation, LUHMES cells (15) were kept on flasks (Nunc, Thermo Fisher Scientific, Waltham, MA, USA), coated with poly-L-ornithine (0.1 mg/ml; 4°C, overnight; Sigma-Aldrich, St. Louis, MO, USA) in a DMEM/F12 medium (Sigma-Aldrich, St. Louis, MO, USA) with 1% N2-supplement (Thermo Fisher Scientific) and a 0.04 μg/ml basic fibroblast growth factor (bFGF, PeproTech, Rocky Hill, CT, USA). For differentiation, the cells were seeded in a differentiation medium at a density of 100,000 cells per cm^2^ on cell culture dishes coated with poly-L-ornithine (0.1 mg/ml; 4°C, overnight; Sigma-Aldrich), followed by coating with fibronection (5 μg/ml, 37°C, overnight; Sigma-Aldrich). The differentiation medium consisted of a DMEM/F12 medium with 1% N2-supplement, 1-μg/ml tetracycline (Sigma-Aldrich), 0.49-μg/ml dibutyryl cyclic adenosine monophosphate, and a 2-ng/ml glial cell-derived neurotrophic factor (GDNF, R&D Systems, Minneapolis, MN, USA).

### Adenoviral Transduction

For the experiments, only cells from passages two to four were taken. For all experiments, cells were seeded independently. Two days after plating (i.e., starting of the differentiation process), adenoviral vectors to overexpress αSyn or green fluorescent protein (GFP; BioFocus, Charles River Laboratories Nederland B.V., Leiden, Netherlands) were added to the cells at a multiplicity of infections of 2, as described previously (17). After 24 h, the remaining adenoviral vectors were removed from the medium by washing the cells three times with PBS (Thermo Fisher Scientic). Thereafter, fresh differentiation medium was added.

### Samples Preparation for Transcriptome Analysis

Four days after transduction (i.e., 6 days into the differentiation process), the cells were washed with PBS, and RLT buffer (Qiagen, Hilden, Germany), activated with β-mercaptoethanol prior to usage, was added to the cells to extract mRNA. Three independent samples from independently seeded cells each from αSyn- and GFP-overexpressing cells were collected for analysis.

### Transcriptome Analysis

The expression of genes was determined with Illumina Human HT-12 V3 bead chips (Illumina, San Diego, CA, USA). In order to estimate significantly differentially expressed transcripts and their associated enriched functional gene ontology (GO) annotations, the measured data were analyzed. To this end, the data were preprocessed using Illumina Genome Studio Software (v. 1.0.6), including background subtraction and mapping of features to identifiers (GENE Symbol and ENTREZ IDs). The output obtained was further analyzed using **R (Version 4.0.2)** (18) with the integrated development environment **RStudio (Version 1.3.1056)** if not stated otherwise. For data importing, tidying, transforming, and statistical testing, the **tidyverse (1.3.0)** (19) and R base packages were used. Data normalization between arrays and variance-based filtering was performed using **limma (3.44.3)** (20) and **genefilter (1.74.0)**, respectively, with **BiocManager (1.30.10)** to manage Bioconductor packages. Result tables were saved as excel files with **openxlsx (4.1.5)**. In brief, negative values of probe level data from GenomeStudio output were forced positively by applying a minimal fixed intensity offset to all values. Corrected values were log2 transformed for variance stabilization, followed by quantile normalization. Next, feature intensities were filtered based on the detection *p*-values provided by Illumina, i.e., only probes having at least one detection *p*-value ≤ 0.05 were kept. Features, which were not mappable to ENTREZ identifiers, were removed. The experiment-specific-background transciptome was constructed by summarization of the remaining features on ENTREZ ID and the GENE Symbol level. GENE Symbols with multiple ENTREZ IDs were excluded. Expression values that did not show enough variation to allow reliable detection of differential expression were removed using an unspecific variance-based filter on a probe level, where the inter quantile range (IQR) was used as a measure for dispersion, and the 0.5 quantile of the IQR values has been used as a cutoff for removal of uninformative features. Subsequently, features were summarized on ENTREZ ID and the GENE Symbol level based on the normalized median intensities for the respective probes and filtered as described above. Both experimental groups for each feature were compared using a two-sided unpaired *t*-test. In order to control the rate of type I errors, when conducting multiple *t*-tests, *p*-values were adjusted using the Benjamini-Hochberg procedure (21). Features with an adjusted *p*-value < 0.05 were considered as differently expressed between both groups.

### Sample Preparation for Proteome Analysis

For the proteome analysis, samples from cells 4 days after transduction (i.e., 6 days into the differentiation process) were collected. First, the cells were washed two times with PBS supplemented with a protease inhibitor (cOmplete Protease Inhibitor Cocktail (Roche, Basel, Switzerland) and homogenized in a lysis buffer, consisting of 7-M urea, 2-M thiourea, 20-mM tris base, and 4% 3-[(3-cholamidopropyl) dimethylammonio]-1-propanesulfonate (CHAPS) at a pH of 8.5, using a cell scraper. To remove cell debris and crude impurities, the lysates were centrifuged for 15 min at 16.000 × g. Protein concentrations of the supernatants were determined using the Bradford assay (Bio-Rad, Hercules, CA). Nine independent samples from independently seeded cells each from αSyn- and GFP-overexpressing cells were collected for analysis. Prior to LC–MS/MS analysis, we had performed Western blots to confirm successful overexpression of an αSyn or GFP, respectively. The Western blot is shown in Supplementary Figure 1.

### Protein Digestion

About 10 μg proteins per sample were used for tryptic digestion. The urea concentration was lowered to 1.5 M by adding 50-mM ammonium bicarbonate. Samples were digested with 1:28 (w/w) trypsin (SERVA Electrophoresis, Heidelberg, Germany) for 14 h at 37°C. Finally, the peptide concentrations were determined by quantitative amino acid analysis (AAA).

### LC–MS/MS Analysis

LC–MS/MS analysis was performed on a QExactive HF Orbitrap mass spectrometer (Thermo Fisher Scientific), coupled to an Ultimate 3000 RSLCnano HPLC system (Dionex, Thermo Fisher Scientific). All peptide samples were diluted in 15 μL of 0.1% TFA, and 200 ng per proteome sample was loaded and concentrated on a trap column (Acclaim PepMap, 100, 100 μm × 2 cm, nanoViper, C18, 5 μm, 100 Å, Thermo Fisher Scientific) within 7 min at a flow rate of 30 μL/min with 0.1% TFA. Peptide separation was performed on an analytical column (Acclaim PepMap RSLC, 75 μm × 50 cm, nanoViper, C18, 2 μm, 100 Å) at a flow rate of 400 nL/min with a 135-min segmented linear gradient, with a monotonic increase, adapted to the quantity of eluting peptides, from 5 to 52% solvent B (solvent A: 0.1% FA, solvent B: 0.1% FA, 84% acetonitrile). Peptides eluting from the column were ionized at 1.55 kV in the Nanospray Flex Ion Source (Proxeon Biosystems A/S, Odense, Denmark). Survey scans were acquired from 350 to 1,100 m/z at a resolution of 120,000. Data-dependent acquisition of MS/MS spectra was performed with higher-energy collisional dissociation for the 10 highest abundant precursor peptide ions with a resolution of 30,000 and an isolation window = 1.6 m/z. Dynamic exclusion was set to 20 s. The normalized stepped collision energy (NCE) and fixed first mass were set to 24, 27, 30, and 100 m/z, respectively.

### MS Raw Data Processing, Peptide Identification, and Protein Quantification

The generated ^*^.raw files were converted to ^*^.mzML files using the msconvert tool (default settings with the additional filter setting peakPicking and vender msLevel set to 1-1) from ProteWizard (v3.0.0002) (22) and processed with an in-house openMS (v2.6.0) (23) workflow implemented in the free and open-source KNIME data analytics platform (v4.3.1).

The openMS workflow using PIA in combination with quantification was adapted from KNIME hub repository (https://hub.knime.com/julianu/spaces/PIA/latest/02-PIA_and_quant~tjlaE-J0RnMY2LrA). In brief, MS/MS spectra were searched with the database search engine node ***MS-GF**+* (22) against a combined target-decoy database, including the complete human UniProtKB set, potential contaminants (cRAP) and GFP sequences. For the database search, precursor mass tolerance was set to 10 ppm with Trypsin/P and full digestion as an enzyme setup. Methionine (M) oxidation was included as variable modification. A maximum number of missed cleavages were set to one. Protein inference was performed using the advanced protein inference algorithm (PIA) (24, 25) implemented in the corresponding ***PIA*** node. The inference method was set to an spectrum extractor with a PSM score filter set to 0.01. The scoring method was set to multiplicative scoring, and only the best PSM score per peptide was used. For quantification, individual peptide features (MS1 level) were determined using the ***FeatureFinderMultiplex*** node in a label-free mode. Identified peptides were combined and mapped to the peptide features using the ***PIA*** node on the peptide spectrum matches (PSMs) level and the ***IDMapper*** node, respectively. False discovery rate (FDR) thresholds were set to 0.01 on peptide and protein levels using ***IDFilter*** and ***PIA*** nodes, respectively. Mapped features were aligned by a retention time correction between feature maps using the ***MapAlignerIdentifications*** node. Groups of corresponding features from multiple maps were linked *via* the ***FeatureLinkerUnlabeledQT*** node and, subsequently, quantile normalized with the ***ConsensusMapNormalizer*** node. Ambiguous annotations with features with peptide identifications were resolved using the ***IDConflictResolver*** node. Finally, protein and peptide abundances were computed using the ***ProteinQuantifier*** node, considering all charge states and all peptides assigned to a protein based on the PIA results. Here, protein abundances were computed based on median abundances of the three most abundant proteotypic peptides. Results were exported as text files and further processed using R. The mass spectrometry realted proteomics data have been deposited to the ProteomeXchange Consortium *via* the PRIDE (26) partner repository with the dataset identifier PXD028322 and 10.6019/PXD028322.

### Proteomics Data Analysis

Data analysis was performed using KNIME and R (compare data analysis for transcriptomics). In brief, contaminants and decoy hits were filtered out for proteomics data. Only protein groups (in the following, referred to as proteins) with ≥2 peptides in total were considered for analysis. Intensity values containing zeros were replaced with NA. Remaining intensities were log2-transformed. Protein groups with at least one valid value in at least one sample were considered as identified and, in the following, used to construct the proteome background set. To allow a comparison and integration with the transciptome data, UNIPROT ACCESSIONS listed within each protein group were mapped to ENTREZ and GENE SYMBOL IDs using the bioconductor homo sapiens annotation package **org.Hs.eg.db** (**3.12.0**). ENTREZ IDs were used as a primary mapping identifier. Subsequentially, protein groups were updated as follows: first UNIPROT ACCESSIONS with no matching ENTREZ ID were removed as well as any resulting empty protein group. In addition, 33 UNIPROT ACCESSIONS, which had GENE SYMBOLS mappable to multiple ENTREZ IDs, were removed. No entries were found where an ENTREZ ID was mappable to multiple GENE SYMBOLS. However, there was one ENTREZ ID found, which was mappable to two different protein groups. Both protein groups and the corresponding ENTREZ ID were consequentially removed from the data. The resulting mappable list of protein groups represented the experiment-specific proteome background. For further statistical data analysis, only entries (protein groups), which had at least two valid intensity values in each experimental group, were considered. Analogous to transcriptome data analysis, both experimental groups for each protein group were compared using a two-sided unpaired *t*-test. In order to control the rate of type I errors, when conducting multiple *t*-tests, *p*-values were adjusted using the Benjamini-Hochberg procedure (21). Features with an adjusted *p*-value < 0.05 were considered as differently expressed between both groups.

### Western Blot Analyses

For Western blot analyses, samples from cells 4 days after transduction (i.e., 6 days into the differentiation process) with αSyn- or GFP-expressing viral vectors or untransduced cells 6 days into the differentiation process were collected using an M-PER buffer (Thermo Fisher Scientific), supplemented with a protease inhibitor (cOmplete Protease Inhibitor Cocktail (Roche, Basel, Switzerland). For electrophoresis, the samples were loaded on Criterion TX or TGX gels (Bio-Rad) with 20 μg protein per lane. After electrophoresis, the proteins were blotted on PVDF membranes, followed by blocking with 5% skim milk in Tris-buffered saline, containing 0.05% Tween 20 (TBS-T, pH 7.4). For visualization of the proteins, the following primary antibodies were used: rabbit anti-ADD1 (1:500; Abcam, Cambridge United Kingdom), rabbit anti-ALDH6A1 (1:3,000; Proteintech, Rosemont, IL, USA), rabbit anti-ARHGEF2 (1:1,000; Cell Signaling Technology, Inc., Danvers, MA, USA), rabbit anti-ASNS (1:500; Proteintech), rabbit anti-αSyn (1:500; Thermo Fisher Scientific), goat anti-CNTN2 [1 μg/ml (R&D Systems, Minneapolis, MN, USA)], mouse anti-DLG4/PSD95 (1:500; Synaptic Systems, Göttingen, Germany), rabbit anti-EDF1 (1:500; Proteintech), mouse anti-LHX9 (1:500; Santa Cruz Biotechnology, Dallas, TX, USA), sheep anti-MDGA1 (0.1 μg/ml; R&D Systems), rabbit anti MTHFD2 (1:500; Proteintech), rabbit anti-NCALD (1:1,000; Proteintech), rabbit anti-PSAT1 (1:1,000; Proteintech), rabbit anti-SCARB2/LIMP2 (1:1,000; Novus Biologicals; Littleton, CO, USA), rabbit anti-SCG2 (1:1,000; Abcam), rabbit anti-SERPINB6 (1:500; Proteintech), rabbit anti-SHMT2 (1:1,000; Proteintech), rabbit anti-SLC7A1 (1:500; Proteintech), rabbit anti-STXBP1 (1:1,000; Synaptic Systems), mouse anti-TFRC (1:1,000; Thermo Fisher Scientific), rabbit anti-WARS1 (1:500; Thermo Fisher Scientific). After three-time washing with TBS-T, the membranes were incubated with a horseradish peroxidase (HRP)-conjugated secondary antibody (1:5,000; Vector Laboratories). For image acquisition, the Odyssey Fc (LI-COR Biotechnology, Lincoln, NE, USA) imaging system was used after incubation with Clarity Western ECL Substrate (Bio-Rad). As loading control, we used a rabbit anti-β-Actin antibody conjugated with HRP (1:2,000; Cell Signaling Technology), or an anti-rabbit glyceraldehyde 3-phosphate dehydrogenase (GAPDH) antibody (1:1,000, Cell Signaling Technology). For quantification, the densities of the bands were normalized to the respective β-Actin or GAPDH bands.

### Network and Enrichment Analysis

The differentially expressed genes and proteins were imported into Cytoscape 3.8.2 (64 bit version) for Windows (27). The interaction and enrichment analyses were performed using the stringApp for Cytoscape with resources from StringDB (version 11.0) (28). Networks of full network type were generated, considering only high confidence interactions (interaction score 0.7). Otherwise, the standard settings were used. Enriched Gene Ontologies, KEGG, Reactome pathways, and string interactions were exported and further analyzed.

## Results

### Differentially Expressed Genes and Proteins Between αSyn-Overexpressing and GFP-Expressing LUHMES Cells

**Figure 1:**
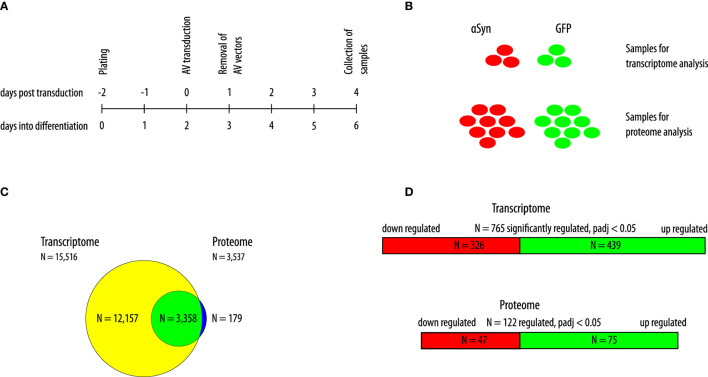
Description of study design and samples. **(A)** Schematic of the experimental design. Cells were transduced 2 days into the differentiation process and collected 4 days after transduction. AV, adenoviral vectors. The upper timeline indicates days post transduction; the lower timeline indicates days into the differentiation process. **(B)** In total, three samples of αSyn-overexpressing cells and GFP-expressing cells were collected for the transcriptome analysis, and nine samples of αSyn-overexpressing cells and GFP-expressing cells were collected for the proteome analysis. **(C)** Overlap between detected genes in the transcriptome analysis and corresponding proteins in the proteome analysis. *N* = 15,516 transcripts were detected and 3,537 proteins. The yellow circle shows items exclusively detected in the transcriptome (*N* = 12,157). The green circle indicates items detected in both, the transcriptome and the proteome (*N* = 3,358). The blue circle indicates items exclusively detected in the proteome (*N* = 179). **(D)** Depiction of differentially regulated transcripts (an upper panel) and proteins (a lower panel) with an adjusted *p*-value < 0.05 (Benjamini-Hochberg false-discovery rate). Red indicates downregulation. Green indicates upregulation.

**Figure 2:**
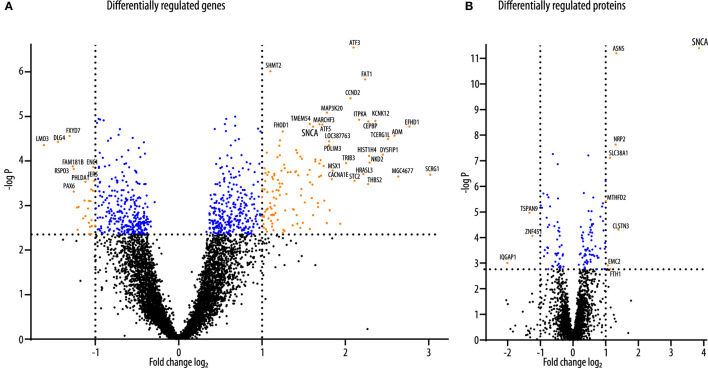
Volcano plots of differentially regulated genes and proteins. **(A)** The volcano plot of transcripts of genes that were differentially regulated between αSyn-overexpressing and GFP-expressing LUHMES cells. Blue dots indicate all transcripts that were above the threshold for statistical significance after correction for multiple testings (adjusted *p*-value < 0.05). Orange dots indicate those of the statistically significant regulated transcripts were regulated more than 2-fold. Of these, 27 were downregulated (orange dots on the left), and 130 were upregulated (orange dots on the right). **(B)** The volcano plot of proteins that were differentially regulated between αSyn-overexpressing and GFP-expressing LUHMES cells. Blue dots indicate all proteins that were above the threshold for statistical significance after correction for multiple testings (adjusted *p*-value < 0.05). Orange dots indicate those of the statistically significant regulated proteins that were regulated more than 2-fold. Of these, three were downregulated (orange dots on the left), and eight were upregulated (orange dots on the right).

As previously described, LUHMES cells were plated in a differentiation medium and transduced with adenoviral vectors to overexpress human wild-type αSyn or GFP 2 days thereafter (17). We previously showed that LUHMES cells were fully differentiated into a dopaminergic phenotype after 6 days of differentiation. Furthermore, we previously showed that αSyn overexpressing cells show marked toxicity 6 days after transduction when transduction was performed 2 days into the differentiation process, whereas 4 days after transduction (i.e., 6 days into the differentiation process), no toxicity was observed (16). Therefore, in the present study, we collected the cell samples for the analysis of the transcriptome and the proteome 4 days post transduction (i.e., 6 days into the differentiation process). By doing so, we could observe changes in the proteome and transcriptome that were induced as a reaction to the overexpression of αSyn that preceded cell death. Prior to the proteomics analysis, successful overexpression of αSyn or GFP, respectively, was confirmed by Western blot analysis (Supplementary Figure 1). The experimental design is shown in Figure 1A. For the analysis of the transcriptome, *N* = 3 samples of each group were analyzed. For the analysis of the proteome, *N* = 9 samples of each group were analyzed (Figure 1B). In total, we detected *N* = 15,516 transcripts in the transcriptome background and *N* = 3,537 proteins in the proteome background. Most of the proteins that were detected in LUHMES cells (*N* = 3,358; 95.0%) were also present in the transcriptome dataset. *N* = 12,157 transcripts were detected without the corresponding protein, and *N* = 179 proteins were detected without the corresponding transcript (Figure 1C). We then performed a filtering step before analyzing the transcriptome dataset (see Methods). After filtering, 8,609 genes were further analyzed. Of these genes, *N* = 765 genes were significantly differentially regulated between αSyn-overexpressing and GFP-expressing LUHMES cells (adjusted *p* < 0.05; Benjamini-Hochberg FDR). Of these, *N* = 439 were upregulated and *N* = 326 were downregulated (Figure 1D, upper panel). In the proteome dataset, we analyzed all proteins that were detected (*N* = 3,538). We found that *N* = 122 proteins were differentially regulated between the αSyn-overexpressing and the GFP-expressing LUHMES cells (adjusted *p* < 0.05; Benjamini-Hochberg FDR). Of these, *N* = 75 proteins were upregulated, and *N* = 47 proteins were downregulated (Figure 1D lower panel). Of the statistically significantly regulated genes, 130 were upregulated at least 2-fold, and 27 were downregulated at least 2-fold (Figure 2A). Eight of the statistically significantly regulated proteins were at least 2-fold (log_2_ > 1) upregulated, and three were at least 2-fold (log_2_ < −1) downregulated (Figure 2B). Furthermore, we ranked the genes and proteins that were most strongly regulated between αSyn-overexpressing and GFP-expressing cells according to the Euclidian distance; a mathematical parameter that takes the degree of regulation and the *p*-value into account was used to rank. The list of the top 50 genes that were most strongly regulated is shown in Table 1; the list of the top 50 proteins that were most strongly regulated is shown in Table 2. Expectantly, αSyn, encoded by *SNCA* (synuclein alpha) was the most strongly regulated protein in the proteome dataset with a Euclidian distance of 12 (p = 1.1e-8, Benjamini-Hochberg FDR).

**Table 1:** Top 50 differentially regulated transcripts between alpha-synuclein-overexpressing and GFP-expressing cells, sorted by Euclidian distance.

**Gene symbol**	**Gene name**	**Log_**2**_FC**	**P_**adj**_**	**Euclid distance**	**Direction**
*ATF3*	Activating transcription factor 3	2.098	0.002	6.877	Up
*FAT1*	FAT atypical cadherin 1	2.238	0.004	6.246	Up
*SHMT2*	Serine hydroxymethyltransferase 2	1.101	0.004	6.115	Up
*CCND2*	Cyclin D2	2.060	0.007	5.787	Up
*EFHD1*	EF-hand domain family member D1	2.770	0.007	5.519	Up
*KCNK12*	Potassium two pore domain channel subfamily K member 12	2.360	0.007	5.440	Up
*CEBPB*	CCAAT Enhancer Binding Protein Beta	2.275	0.007	5.389	Up
*MAP3K20*	Mitogen-activated protein kinase kinasekinase 20	1.778	0.007	5.388	Up
*ITPKA*	Inositol-trisphosphate 3-kinase A	2.166	0.007	5.380	Up
*ADM*	Adrenomedullin	2.589	0.008	5.251	Up
*TCERG1L*	Transcription elongation regulator 1 like	2.511	0.008	5.146	Up
*ATF5*	Activating transcription factor 5	1.725	0.007	5.116	Up
*MARCHF3*	Membrane associated ring-CH-type finger 3	1.689	0.007	5.111	Up
*TMEM54*	Transmembrane protein 54	1.573	0.007	5.084	Up
*SOX18*	SRY-box transcription factor 18	0.677	0.007	5.042	Up
*MARF1*	Meiosis regulator and mRNA stability factor 1	−0.962	0.007	5.038	Down
*SNCA*	Synuclein alpha	1.612	0.007	5.034	Up
*LRRN3*	Leucine rich repeat neuronal 3	−0.944	0.007	5.020	Down
*RUNX1T1*	RUNX1 partner transcriptional co-repressor 1	−0.902	0.007	4.995	Down
*MKX*	Mohawk homeobox	0.570	0.007	4.978	Up
*CFAP91*	Cilia and flagella associated protein 91	−0.982	0.007	4.953	Down
*AARS1*	Alanyl-tRNA synthetase 1	0.555	0.007	4.860	Up
*PEA15*	Proliferation and apoptosis adaptor protein 15	0.677	0.007	4.839	Up
*FHOD1*	Formin homology 2 domain containing 1	1.248	0.007	4.828	Up
*SLC50A1*	Solute carrier family 50 member 1	0.952	0.007	4.822	Up
*PPP1R27*	Protein phosphatase 1 regulatory subunit 27	2.448	0.012	4.812	Up
*C11orf96*	Chromosome 11 open reading frame 96	1.805	0.008	4.795	Up
*UBXN11*	UBX domain protein 11	−0.706	0.007	4.768	Down
*SCRG1*	Stimulator of chondrogenesis 1	3.019	0.014	4.768	Up
*BAIAP2L1*	BAR/IMD domain containing adaptor protein 2 like 1	0.980	0.007	4.767	Up
*FXYD7*	FXYD domain containing ion transport regulator 7	−1.309	0.008	4.745	Down
*KCTD5*	Potassium channel tetramerization domain containing 5	0.432	0.007	4.739	Up
*PDLIM3*	PDZ and LIM domain 3	1.802	0.009	4.711	Up
*SBF1*	SET binding factor 1	0.565	0.007	4.705	Up
*H4C8*	H4 clustered histone 8	2.283	0.012	4.705	Up
*DLG4*	Discs large MAGUK scaffold protein 4	−1.449	0.008	4.658	Down
*LMO3*	LIM domain only 3	−1.618	0.009	4.648	Down
*SLC7A6*	Solute carrier family 7 member 6	−0.721	0.008	4.644	Down
*SBSPON*	Somatomedin B and thrombospondin type 1 domain containing	1.215	0.008	4.624	Up
*RPRD1A*	Regulation of nuclear pre-mRNA domain containing 1A	−0.815	0.008	4.585	Down
*NKD2*	NKD inhibitor of WNT signaling pathway 2	2.290	0.013	4.578	Up
*LINC01091*	Long intergenic non-protein coding RNA 1091	−0.871	0.008	4.573	Down
*CTNNAL1*	Catenin alpha like 1	0.721	0.008	4.552	Up
*FBXO33*	F-box protein 33	−0.553	0.008	4.550	Down
*NTF3*	Neurotrophin 3	−0.849	0.008	4.532	Down
*PALLD*	Palladin, cytoskeletal associated protein	0.679	0.008	4.513	Up
*BCAT1*	Branched chain amino acid transaminase 1	1.206	0.009	4.509	Up
*CYTOR*	Cytoskeleton regulator RNA	2.636	0.015	4.502	Up
*DDIT3*	DNA damage inducible transcript 3	1.411	0.010	4.473	Up
*TTLL7*	Tubulin tyrosine ligase like 7	−0.419	0.008	4.439	Down

**Table 2:** Top 50 differentially regulated proteins between alpha-synuclein-overexpressing and GFP-expressing cells, sorted by Euclidian distance.

**Gene symbol**	**Gene name**	**Log_**2**_FC**	**P_**adj**_**	**Euclid distance**	**Direction**
*SNCA*	Synuclein alpha	3.856	1.1e-8	12.037	Up
*ASNS*	Asparagine synthetase (glutamine-hydrolyzing)	1.326	1.1e-8	11.281	Up
*NRP2*	Neuropilin 2	1.308	2.73e-5	7.742	Up
*STXBP1*	Syntaxin binding protein 1	−0.616	4.36e-5	7.287	Down
*NES*	Nestin	0.468	4.36e-5	7.221	Up
*SLC38A1*	Solute carrier family 38 member 1	1.135	4.4e-5	7.210	Up
*SPTBN1*	Spectrin beta, non-erythrocytic 1	−0.468	6.8e-5	6.886	Down
*SLC1A4*	Solute carrier family 1 member 4	0.996	2.7e-4	6.290	Up
*RPL26*	Ribosomal protein L26	−6.072	0.624	6.112	Down
*LHX9*	LIM homeobox 9	−0.907	0.001	5.792	Down
*PITPNM1*	Phosphatidylinositol transfer protein membrane associated 1	0.329	0.001	5.751	Up
*BRD3*	Bromodomain containing 3	−0.790	0.001	5.730	Down
*SUSD2*	Sushi domain containing 2	−0.751	0.001	5.621	Down
*ABCB6*	ATP binding cassette subfamily B member 6 (Langereis blood group)	0.623	0.001	5.597	Up
*EPB41L5*	Erythrocyte membrane protein band 4.1 like 5	−0.458	0.001	5.551	Down
*MTHFD2*	Methylenetetrahydrofolate dehydrogenase (NADP+ dependent) 2	1.009	0.001	5.520	Up
*LGALS3BP*	Galectin 3 binding protein	0.916	0.001	5.420	Up
*C2CD5*	C2 calcium dependent domain containing 5	−0.986	0.001	5.346	Down
*SMPD1*	Sphingomyelin phosphodiesterase 1	0.895	0.001	5.292	Up
*FAM171A1*	Family with sequence similarity 171 member A1	−0.700	0.001	5.266	Down
*NDRG1*	N-myc downstream regulated 1	−0.951	0.001	5.249	Down
*TSPAN9*	Tetraspanin 9	−1.333	0.002	5.145	Down
*SLC7A1*	Solute carrier family 7 member 1	0.569	0.001	5.062	Up
*STON2*	Stonin 2	0.478	0.001	5.058	Up
*ABCA3*	ATP binding cassette subfamily A member 3	0.842	0.002	4.892	Up
*CNTN2*	Contactin 2	−0.807	0.002	4.842	Down
*TFRC*	Transferrin receptor	0.914	0.003	4.740	Up
*LINGO1*	Leucine rich repeat and Ig domain containing 1	0.541	0.003	4.728	Up
*CELF1*	CUGBP Elav-like family member 1	−0.471	0.003	4.633	Down
*SCARB2*	Scavenger receptor class B member 2	0.616	0.004	4.575	Up
*RRM1*	Ribonucleotide reductase catalytic subunit M1	0.580	0.004	4.561	Up
*FIBP*	FGF1 intracellular binding protein	−0.728	0.004	4.555	Down
*CLSTN3*	Calsyntenin 3	1.391	0.004	4.531	Up
*NEFL*	Neurofilament light chain	0.579	0.004	4.506	Up
*STX1B*	Syntaxin 1B	−0.570	0.004	4.485	Down
*ALDH6A1*	Aldehyde dehydrogenase 6 family member A1	−0.773	0.004	4.480	Down
*ESYT1*	Extended synaptotagmin 1	0.528	0.004	4.432	Up
*TXNDC5*	Thioredoxin domain containing 5	0.241	0.004	4.425	Up
*XRCC6*	X-ray repair cross complementing 6	−0.428	0.004	4.405	Down
*TTYH3*	Tweety family member 3	0.933	0.005	4.381	Up
*ARL15*	ADP ribosylation factor like GTPase 15	0.547	0.004	4.338	Up
*SPTBN2*	Spectrin beta, non-erythrocytic 2	−0.457	0.005	4.295	Down
*PHC2*	Polyhomeotic homolog 2	−0.415	0.005	4.252	Down
*ZNF451*	Zinc finger protein 451	−1.244	0.006	4.251	Down
*KHDRBS3*	KH RNA binding domain containing, signal transduction associated 3	0.525	0.005	4.246	Up
*DAGLB*	Diacylglycerol lipase beta	0.590	0.005	4.234	Up
*IARS1*	Isoleucyl-tRNA synthetase 1	0.311	0.005	4.219	Up
*PLD3*	Phospholipase D family member 3	0.478	0.006	4.148	Up
*EPRS1*	Glutamyl-prolyl-tRNA synthetase 1	0.273	0.006	4.099	Up
*PLXNA3*	Plexin A3	−0.407	0.006	4.095	Down

### Overlap Between Genes and Proteins in the Transcriptome and the Proteome Dataset

**Figure 3:**
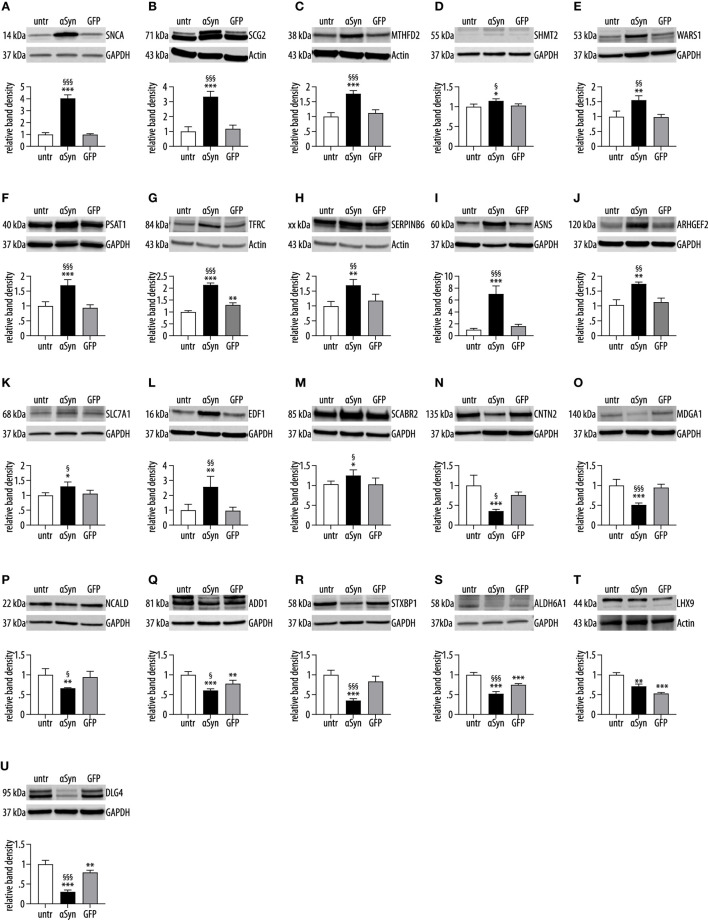
Western blots confirmation. Western blots with antibodies against the 21 proteins that were differentially regulated in the proteomics, of which the corresponding genes were also differentially regulated. For the Western blot investigation, samples from untransduced cells (untr), αSyn-overexpressing cells (αSyn), and GFP-expressing cells (GFP) cells were used. The proteins are presented in the same order as in Table 3. **(A)** SNCA, synuclein alpha; **(B)** SCG2, Secretogranin II; (C) MTHFD2, methylenetetrahydrofolate dehydrogenase (NADP+ dependent) 2, methenyltetrahydrofolate; **(D)** SHMT2, serine hydroxymethyltransferase 2; **(E)** WARS1, tryptophanyl-tRNA synthetase 1; **(F)** PSAT1, phosphoserine aminotransferase 1; **(G)** TFRC, transferrin receptor; **(H)** SERPINB6, serpin Family B, Member 6; **(I)** ASNS, asparagine synthetase (glutamine-hydrolyzing); **(J)** ARHGEF2, Rho/Rac guanine nucleotide exchange Factor 2; **(K)** SLC7A1, solute carrier Family 7, Member 1; **(L)** EDF1, endothelial differentiation-related Factor 1; **(M)** SCARB2, scavenger receptor Class B, Member 2; **(N)** CNTN2, contactin 2; **(O)** MDGA1, MAM domain containing glycosylphosphatidylinositol Anchor 1; **(P)** NCALD, neurocalcin delta; **(Q)** ADD1, Adducin 1; **(R)** STXBP1, syntaxin-binding Protein 1; **(S)** ALDH6A1, aldehyde dehydrogenase 6 Family, Member A1; **(T)**: LHX9, LIM Homeobox 9; **(U)** DLG4, discs large MAGUK scaffold Protein 4. Twenty out of 21 proteins **(A–S,U)** were differentially regulated between αSyn-overexpressing cells and GFP-expressing cells in the same direction as in the LC-MS investigation and thereby confirming these findings. With the exception of TFRC **(G)**, ADD1 **(Q)**, ALDH6A1 **(S)**, and DLG4 **(U)**, there was no difference in protein levels between untransduced cells and GFP-expressing cells, suggesting an αSyn-specific effect of the protein regulation. One protein (LHX9, **T**) could not be confirmed. *p < 0.05, **p < 0.01, ***p < 0.001 vs. untransduced cells. ^§^*p* < 0.05, ^§§^*p* < 0.01, ^§§§^*p* < 0.001 vs. GFP transduced cells.

Of the *N* = 122 proteins and *N* = 765 genes that were differentially regulated between αSyn and GFP expressing cells, *N* = 21 were significantly regulated in both the transcriptome and the proteome. All of these 21 genes/proteins were regulated in the same direction. About 13 genes/proteins were upregulated, and eight were downregulated. Among the 21 genes/proteins that were regulated on the proteome and the transcriptome level, eight were associated with synapses, including *DLG4* (discs large MAGUK scaffold protein 4), the gene encoding for the synaptic protein disks large homolog 4, also known as postsynaptic density protein 95 (PSD-95). Four of the genes/proteins (mitochondrial serine hydroxymethyltransferase encoded by *SHMT2* [serine hydroxymethyltransferase 2], phosphoserine aminotransferase, encoded by *PSAT1* [phosphoserine aminotransferase 1], asparagine synthetase, encoded by *ASNS* [asparagine synthetase (glutamine-hydrolyzing)], and high-affinity cationic amino acid transporter 1, encoded by *SLC7A1* [solute carrier family 7, member 1]) are involved in amino acid metabolism or transport. Three genes/proteins are involved in mitochondrial function: bifunctional methylenetetrahydrofolate dehydrogenase, encoded by *MTHFD2* [methylenetetrahydrofolate dehydrogenase (NADP + dependent) 2], methylmalonate-semialdehyde dehydrogenase, encoded by *ALDH6A1* [aldehyde dehydrogenase 6 family member A1], and mitochondrial serine hydroxymethyltransferase, encoded by *SHMT2*; one-protein LIMP2 - Lysosome membrane protein 2 encoded by *SCARB2* [scavenger receptor Class B, Member 2] is involved in lysosomal function. The list of the 21 genes/proteins that were significantly regulated in the transcriptome and proteome is shown in Table 3. The upregulation on the proteome level of these 21 proteins was also investigated in αSyn-overexpressing, GFP-overexpressing and untransduced control cells by Western blot analyses. All but one (LHX9) of the 21 proteins were significantly regulated between αSyn and GFP overexpressing cells in the same way as in the proteome analysis, whereas most genes were not regulated between GFP overexpressing and untransduced control cells, indicating that the observed regulatory changes were specific for αSyn overexpression (Figure 3).

**Table 3:** Regulation of the 21 transcripts/proteins that were differentially regulated between alpha-synuclein-overexpressing and GFP-expressing cells in the transcriptomic and proteomic level.

**Gene symbol**	**Gene name**	**Differential regulation in transcriptome**		**Differential regulation in proteome**		**Keyword**
		**Log_**2**_FC**	**P_**adj**_**	**Log_**2**_FC**	**P_**adj**_**	
*SNCA*	Synuclein Alpha	1.612	0.007	3.855	1.1e-8	
*SCG2*	Secretogranin II	1.399	0.014	0.736	0.02	Synapse
*MTHFD2*	Methylenetetrahydrofolate Dehydrogenase (NADP+ Dependent) 2, Methenyltetrahydrofolate	1.108	0.039	1.009	8.7e-4	Mitochondrial
*SHMT2*	Serine Hydroxymethyltransferase 2	1.101	0.004	0.45	0.031	Amino acid metabolism
*WARS1*	Tryptophanyl-tRNA Synthetase 1	1.068	0.03	0.57	0.02	tRNA-Sythase
*PSAT1*	Phosphoserine Aminotransferase 1	1.041	0.014	0.504	0.016	Amino acid metabolism
*TFRC*	Transferrin Receptor	0.967	0.043	0.914	0.003	endocytosis
*SERPINB6*	Serpin Family B Member 6	0.911	0.014	0.436	0.035	serine proteinase inhibitor
*ASNS*	Asparagine Synthetase (Glutamine-Hydrolyzing)	0.874	0.022	1.326	1.1e-8	Amino acid metabolism
*ARHGEF2*	Rho/Rac Guanine Nucleotide Exchange Factor 2	0.815	0.013	0.654	0.02	Synapse
*SLC7A1*	Solute Carrier Family 7 Member 1	0.777	0.032	0.569	0.001	Amino acid transport
*EDF1*	Endothelial Differentiation Related Factor 1	0.575	0.048	0.393	0.016	Transcription factor
*SCARB2*	Scavenger Receptor Class B Member 2	0.401	0.03	0.616	0.004	lysosome
*CNTN2*	Contactin 2	−0.691	0.029	−0.807	0.002	Synapse
*MDGA1*	MAM Domain Containing Glycosylphosphatidylinositol Anchor 1	−0.703	0.032	−0.424	0.044	Synapse
*NCALD*	Neurocalcin Delta	−0.747	0.039	−0.315	0.02	Calcium sensor
*ADD1*	Adducin 1	−0.762	0.037	−0.475	0.039	Synapse
*STXBP1*	Syntaxin Binding Protein 1	−0.833	0.033	−0.616	4.4e-5	Synapse
*ALDH6A1*	Aldehyde Dehydrogenase 6 Family Member A1	−1.147	0.037	−0.773	0.004	Mitochondrial
*LHX9*	LIM Homeobox 9	−1.201	0.029	−0.907	6.6e-4	Transcription factor
*DLG4*	Discs Large MAGUK Scaffold Protein 4	−1.449	0.008	−0.865	0.016	Synapse

### Enrichment Analysis

Furthermore, we used the STRING plugin in Cytoscape to perform gene ontology (GO) enrichment analyses. First, we performed a STRING analysis of all genes and proteins that were regulated in at least one of the datasets. The GO terms with the strongest enrichment were “nervous system development” (*N* = 178; FDR = 3.14e-10), “positive regulation of cellular process” (*N* = 331; FDR = 1.83e-9), and “positive regulation of the biological process” (*N* = 346; FDR = 9.48e-8), all in the category “biological process.” The whole GO analysis of all genes and proteins can be found in the Supplementary Material. Since regulatory responses to a stimulus are first visible in the change of the expression of genes, we first performed an enrichment analysis of the *N* = 765 genes that were significantly regulated in our cell model. The most enriched term was “protein binding” (*N* = 358, FDR = 6.09e-5 in the category “molecular function,” followed by “regulation of the developmental process” (*N* = 162; FDR = 1.5e-4) in the category “biological process.” The 15 most enriched GO terms of the transcriptome are shown in Table 4. The full GO enrichment of the whole transcriptome can be found in Supplementary Table 1. Furthermore, we analyzed the genes that were upregulated and downregulated separately. In the enrichment analysis of the upregulated genes, the terms with the strongest enrichment were in the category “biological process” with “regulation of cell death” (*N* = 71; FDR = 2.66e-71), “regulation of programmed cell death” (*N* = 34; FDR = 2.83e-27), and “regulation of the apoptotic signaling pathway” (*N* = 27; FDR = 1.63e-23), being the most strongly enriched GO terms. In the analysis of the downregulated genes, the top terms were also all in the category “biological process.” These were “nervous system development” (*N* = 80; FDR = 1.21e-6), “neuron differentiation” (*N* = 44; FDR = 3.09e-5), and “neurogenesis” (*N* = 59; FDR = 3.68e-5). In the category “cellular component,” the most enriched terms were “nuclear lumen” (*N* = 124; FDR = 2.44e-6), “nucleoplasm” (*N* = 108; FDR = 4.08e-6), and “nucleus” (*N* = 165; FDR = 3.35e-5). The top GO terms of the upregulated genes are shown in Table 5; the top GO terms of the downregulated genes are shown in Table 6. The whole enrichment analysis of the upregulated genes can be found in the Supplementary Table 2, and the whole enrichment analysis of the downregulated genes can be found in Supplementary Table 3.

**Table 4:** Gene ontology (GO) terms with the strongest enrichment in the analysis of all differentially expressed transcripts in the comparison between alpha-synuclein-overexpressing and GFP-expressing LUHMES cells.

**GO category**	**Description**	**FDR value**	**No of genes**
Molecular Function	Protein binding	6.09E-5	358
Biological Process	Regulation of developmental process	1.5E-4	162
Biological Process	Tube development	2.0E-4	68
Biological Process	Positive regulation of cellular process	2.0E-4	294
Biological Process	System development	2.0E-4	230
Biological Process	Multicellular organism development	2.8E-4	253
Biological Process	Regulation of cell population proliferation	4.4E-4	103
Biological Process	Regulation of multicellular organismal development	4.4E-4	130
Biological Process	Nervous system development	4.6E-4	148
Biological Process	Tube morphogenesis	4.6E-4	54
Biological Process	Positive regulation of biological process	4.6E-4	308
Biological Process	Regulation of multicellular organismal process	4.6E-4	178
Biological Process	Regulation of molecular function	6.0E-4	249
Biological Process	Signaling	6.9E-4	244
Biological Process	Positive regulation of developmental process	7.4E-4	93

**Table 5:** Gene ontology (GO) terms with the strongest enrichment in the analysis of the differentially upregulated transcripts in the comparison between alpha-synuclein-overexpressing and GFP-expressing LUHMES cells.

**GO category**	**Description**	**FDR value**	**No of genes**
Biological Process	Regulation of cell death	2.66E-71	71
Biological Process	Positive regulation of programmed cell death	2.83E-27	34
Biological Process	Regulation of apoptotic signaling pathway	1.63E-23	27
Biological Process	Regulation of multicellular organismal development	2.08E-13	35
Biological Process	Regulation of protein phosphorylation	2.91E-13	30
Biological Process	Negative regulation of cell population proliferation	1.14E-12	22
Biological Process	Regulation of response to stress	1.34E-12	29
Biological Process	Response to oxygen-containing compound	1.44E-12	30
Biological Process	Regulation of extrinsic apoptotic signaling pathway	1.12E-11	13
Biological Process	Cellular response to stress	1.52E-11	30
Biological Process	Apoptotic process	2.23E-11	23
Biological Process	Positive regulation of cell population proliferation	2.25E-11	23
Biological Process	Negative regulation of multicellular organismal process	1.21E-10	25
Biological Process	Positive regulation of transport	1.96E-10	22
Biological Process	Regulation of intrinsic apoptotic signaling pathway	2.69E-10	12

**Table 6:** Gene ontology (GO) terms with the strongest enrichment in the analysis of the differentially downregulated transcripts in the comparison between alpha-synuclein-overexpressing and GFP-expressing LUHMES cells.

**GO category**	**Description**	**FDR value**	**No of genes**
Biological Process	Nervous system development	1.21E-6	80
Cellular component	Nuclear lumen	2.44E-6	124
Cellular component	Nucleoplasm	4.08E-6	108
Biological Process	Neuron differentiation	3.09E-5	44
Cellular component	Nucleus	3.34E-5	165
Biological Process	Neurogenesis	3.68E-5	59
Biological Process	Generation of neurons	4.55E-5	56
Cellular component	Intracellular organelle	7.95E-5	242
Biological Process	Regulation of multicellular organismal development	1.1E-4	67
Cellular component	Intracellular membrane-bounded organelle	1.5E-4	215
Biological Process	Plasma membrane bounded cell projection organization	1.6E-4	44
Cellular component	Intracellular	1.8E-4	264
Cellular component	Intracellular organelle lumen	1.8E-4	135
Biological Process	Central nervous system development	2.6E-4	40
Biological Process	Positive regulation of developmental process	3.9E-4	49

In the analysis of the *N* = 122 significantly regulated proteins, the top GO terms were in the category “cellular component.” The most enriched term was “vesicle” with almost half of the regulated protein (*N* = 59, 48.4%) associated with (FDR = 4.48e-9). Other GO terms in the category “cellular component” that were significantly enriched were “synapse” (*N* = 32 proteins; FDR = 3.7e-8), “postsynapse” (*N* = 18; FDR = 2.36e-5), and “presynapse” (*N* = 15; FDR = 1.8e-4). The most enriched GO terms of the differentially regulated proteome are shown in Table 7. Since “vesicle,” “synapse,” “presynapse,” and “postsynapse” were among the most strongly enriched GO terms in the proteomics data, we also analyzed the proteome data with the SynGo knowledge base for synapse research. This confirmed the overrepresentation of GO terms associated with the synapse (Supplementary Material), suggesting that overexpressing of αSyn in LUHMES cells led to changes at the synapse. Other GO terms in the category “cellular component” that were enriched were “lysosome” (*N* = 17; FDR = 2.4e-4) and “lysosomal lumen” (*N* = 7; FDR = 3.4e-4). Furthermore, “lysosome” was also the only KEGG pathway that showed up in the enrichment analysis of the 122 significantly regulated proteins (*N* = 7; FDR = 0.009, Supplementary Material). Among the GO terms that were significantly enriched in the category “biological process,” the terms “vesicle-mediated transport” (*N* = 30 proteins; FDR = 0.001) and “regulated exocytosis” (*N* = 17; FDR = 0.002) were enriched. The whole enrichment analysis of the proteome can be found in Supplementary Table 4. A separated enrichment analysis of only upregulated proteins can be found in Supplementary Table 5, and a separated enrichment analysis of only downregulated proteins can be found in Supplementary Table 6.

**Table 7:** Gene ontology (GO) terms with the strongest enrichment in the analysis of the differentially proteins in the comparison between alpha-synuclein-overexpressing and GFP-expressing LUHMES cells.

**GO category**	**Description**	**FDR value**	**No of genes**
Cellular Component	Vesicle	4.48E-9	59
Cellular Component	Cell junction	1.27E-8	41
Cellular Component	Synapse	3.7E-8	32
Cellular Component	Extracellular exosome	3.79E-8	40
Cellular Component	Cytoplasmic vesicle	2.61E-7	41
Cellular Component	Plasma membrane region	5.21E-7	28
Cellular Component	Extracellular space	9.94E-7	47
Cellular Component	Plasma membrane	2.25E-6	63
Cellular Component	Neuron projection	4.46E-6	28
Cellular Component	Postsynapse	2.36E-5	18
Cellular Component	Membrane-bounded organelle	4.97E-5	103
Cellular Component	Extracellular region	1.2E-4	50
Molecular Function	Binding	1.4E-4	105
Molecular Function	Protein binding	1.4E-4	74
Cellular Component	Organelle	1.8E-4	107
Cellular Component	Synaptic membrane	1.8E-4	13
Cellular Component	Presynapse	1.8E-4	15
Cellular Component	Secretory vesicle	1.8E-4	21
Cellular Component	Lysosome	2.3E-4	17
Cellular Component	Vacuole	2.7E-4	18
Cellular Component	Membrane	3.0E-4	82
Cellular Component	Axon	3.3E-4	16
Cellular Component	Lysosomal lumen	3.4E-4	7
Cellular Component	Glutamatergic synapse	3.4E-4	12
Cellular Component	Anchoring junction	3.6E-4	18

### Overlap to Previously Published Data

**Figure 4:**
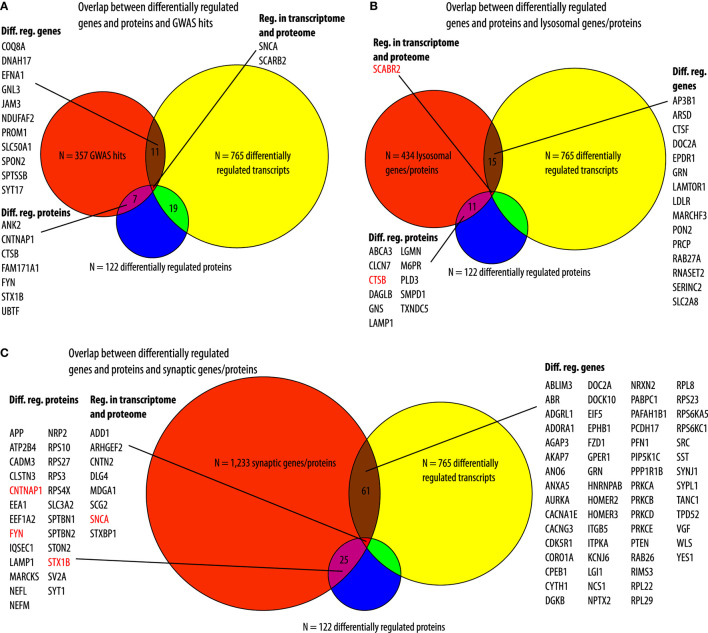
Overlap to existing datasets. **(A)** Overlaps between *N* = 357 genes that were associated with risk loci in two recent Parkinson's disease genome-wide associated studies (GWAS) (an orange circle) and transcripts (a yellow circle) and protein (a blue circle) that were differentially regulated in our cell model. Of the 357 genes associated with PD, *N* = 11 were differentially regulated in our cell model (a brown area). Furthermore, *N* = 7 proteins encoded by these genes were differentially regulated in our cell model (a pink area). In addition to *SNCA* (alpha-synuclein) that was overexpressed in our cells model, also SCARB2 (scavenger receptor Class B, Member 2) was regulated on the transcriptomic and proteomic level (a red area). **(B)** Overlap between *N* = 434 genes present in the human lysosome gene database (an orange circle) and differentially regulated genes (a yellow circle) or proteins (a blue circle) in our cell model. *N* = 15 of the 434 lysosomal genes were differentially regulated in our cell model (a brown area). *N* = 11 proteins that were present in the human lysosome gene database were differentially regulated in our cell model (a pink area). Red characters indicate genes/proteins (SCARB2, scavenger receptor class B, member 2; CTSB, cathepsin B) that were found in the recent GWAS meta-analyses. **(C)** Overlap between *N* = 1,233 synaptic genes (an orange circle) and genes (a yellow circle) or proteins (a blue circle) that were regulated in our cell model. *N* = 61 synaptic genes were differentially regulated in our cell model (a brown area). Furthermore, *N* = 25 of the proteins that were differentially regulated in our cell model were encoded by synaptic genes (a pink area). Red characters mark genes/proteins that have been found in the recent GWAS meta-analyses (FYN, FYN proto-oncogene, Src family tyrosine kinase; STX1B, syntaxin 1B). In addition to SNCA, 7 more genes/proteins were regulated on the transcriptomic and proteomic level in our cell model (a red area). Diff. reg., differentially regulated (adjusted *p*-value < 0.05, Benjanimi-Hochberg false discovery rate).

We compared our datasets with previously published datasets. In a meta-analysis of genome-wide association studies (GWAS), comparing patients with PD and controls, 319 protein-coding genes were identified within 250 kB of associated risk loci (9). In a more recent meta-analysis, loci in 38 more genes have been found (10). We combined these lists and compared the 357 resulting genes to the lists of differentially regulated genes and proteins in our PD cell model. From these 319 genes, 13 were differentially regulated between αSyn overexpressing- and GFP-expressing LUHMES cells on the transcriptome level and 9 on the proteome level. Not surprisingly, *SNCA* was present in all datasets. In addition to *SNCA*, only *SCARB2* (scavenger receptor class B, member 2) was present in the list of PD GWAS hits as well as in the list of regulated genes and proteins in our cell model (Figure 4A). Furthermore, since “lysosome” was among the top GO terms, we compared the list of the differentially regulated genes and proteins with the human lysosome gene database (29). In total, 434 genes/proteins were present in the present lysosomal database. Sixteen of these were overlapping with differentially regulated genes in our cell model, nine were upregulated, and seven were downregulated. Furthermore, of all *N* = 122 proteins that were differentially regulated in our cell model, *N* = 12 proteins (9.8%) were lysosomal proteins, and all of these 12 were upregulated in our cell model. Among these was also, *SCARB2*, which was regulated on the transcriptomic and proteomic level in our cell model as a hit from the recent PD GWAS meta-analyses. Furthermore, also cathepsin B, encoded by *CTSB*, was among the lysosomal proteins that were also present in the hit list from the two recent PD GWAS (Figure 4B). Furthermore, all genes and proteins that were differentially regulated with our cell model were compared to synaptic genes from the SynGo knowledge base for synapse research. *N* = 61 (8.0%) of the *N* = 765 differentially regulated genes and *N* = 25 (20.5%) of the *N* = 122 differentially regulated proteins were associated with synapses. Furthermore, *N* = 8 (38.1%) of the *N* = 21 genes/proteins that were regulated in the transcriptomic and proteomic level were associated with synapses. Moreover, in addition to αSyn, encoded by *SNCA* (synuclein alpha), three other proteins (contactin-associated protein 1, encoded by *CNTNAP1*, tyrosine-protein kinase Fyn, encoded by *FYN*; [FYN proto-oncogene, Src family tyrosine kinase] and syntaxin 1B, encoded by *STX1B*, were also present in the list of genes associated with PD from the GWAS meta-analyses (Figure 4C). Furthermore, independent of the enrichment analysis, we analyzed the presence of genes, which are associated with inflammation and mitochondrial function, in our datasets (see Supplementary Material). Therefore, we searched the Gene Set Enrichment Analysis (GSEA) database for all GO terms associated with inflammation and mitochondria. In total, we identified 28 GO terms associated with inflammation and 42 associated with mitochondria. In total, 796 genes were present in the GO terms associated with inflammation and 837 were present in the GO terms associated with mitochondria. In our model, for 29 of the 765 differentially regulated transcripts and for eight of the 122 differentially regulated proteins, a corresponding gene was associated with inflammation. Of the genes associated with inflammation, SNCA and SCG2 were differentially regulated on the transcriptome and proteome level (Supplementary Figure 2A). Moreover, in our model, for 40 of the 765 differentially regulated transcripts and for 5 of the 122 differentially regulated proteins, a corresponding gene was associated with mitochondria. Of the genes associated with mitochondria, SHMT2, SNCA, and TFRC were differentially regulated on the transcriptome and proteome level (Supplementary Figure 2B).

### Pathway Analysis

**Figure 5:**
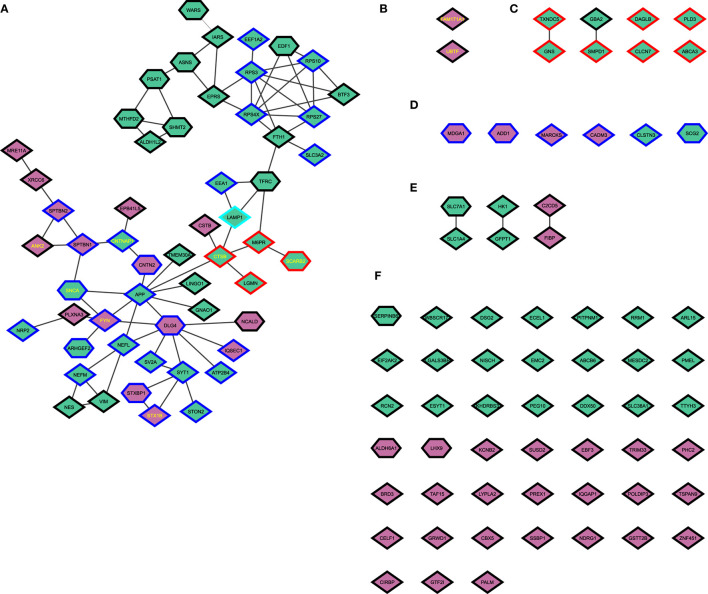
STRING network analysis. **(A)** Interaction network of all differentially regulated proteins with a high confidence level (0.7). **(B)** Protein associated with PD without interactors. **(C)** Proteins associated with the lysosome. **(D)** Proteins associated with synapses. **(E)** Proteins with one interactor. **(F)** Proteins without interactors. Diamonds show proteins only differentially regulated in the proteome. Hexagons indicate proteins with corresponding differentially regulated transcripts. Upregulated proteins are shown in green; downregulated proteins are shown in purple. Red borders indicate proteins with association with the lysosome. Blue borders indicate proteins with association with synapses. A turquois border indicates association with synapses and the lysosome. A yellow label indicates genes that were previously associated with PD in the recent GWAS meta-analyses.

The genes and proteins that were significantly regulated between αSyn-overexpressing and GFP-expressing cells were further analyzed using the STRING plugin in Cytoscape. In total, 55 genes were present in the interaction network of the 122 differentially regulated genes. Of these, 27 were synaptic proteins, of which 19 were upregulated and eight were downregulated. Of all synaptic proteins, in addition to αSyn (*SNCA*), also *CNTNAP1*, encoding contactin-associated protein 1; *FYN* (FYN proto-oncogene, Src family tyrosine kinase), encoding tyrosine-protein kinase Fyn; and *STX1B*, encoding syntaxin 1B, were genes previously associated with PD in GWAS. Furthermore, five of the proteins in the largest interaction network were lysosomal proteins, from which cathepsin B, enconded by *CTSB*, and LIMP2-lysosome membrane protein 2, encoded by *SCARB2* (scavenger receptor class B, member 2) were also associated with PD in the GWAS meta-analyses. Additionally, ankyrin 2, encoded by *ANK2*, an integral membrane protein, was present in the largest interaction network and also associated with PD in the GWAS meta-analyses (Figure 5A). Two other proteins, the genes of which were previously associated with PD (protein FAM171A1), encoded by *FAM171A1* (family with sequence similarity 171, Member A1), and nucleolar transcription factor 1, encoded by *UBTF* (upstream binding transcription factor) had no interactors (Figure 5B). Furthermore, seven further lysosomal proteins had either one or no interactor (Figure 5C), and six further synaptic proteins had no interactor (Figure 5D). Six proteins had only one interactor (Figure 5E), and 45 proteins had no interactors (Figure 5F). Additionally, an interaction of all genes and proteins is shown in Supplementary Figure 3.

## Discussion

In the present study, we performed a transcriptome and proteome analysis in a PD cell model, in which human dopaminergic LUHMES cells (15) show ~50% cell death in the course of 6 days upon moderate adenoviral vector-mediated overexpression of human wild-type αSyn (16). The model is suitable to investigate pharmacological interventions to prevent cell death induced by αSyn overexpression (17, 30). Since we previously showed that there was no cell death present in our model at Day 4 after transduction, while marked cell death of ~50% was observed 6 days after transduction (16), we performed the present transcriptome and proteome analysis with cells at Day 4 after transduction, before cell death occurred. Therefore, we could investigate changes in the transcriptome and proteome that occurred as a consequence of wild-type αSyn overexpression in still vital cells. GFP overexpression was used as control. In total, of 15,516 detected genes, 765 were differentially regulated, and, of 3,537 detected proteins, 122 were differentially regulated between αSyn overexpressing- and GFP-expressing cells. Not surprisingly, αSyn was the strongest regulated protein in our model. In the GO analysis of the upregulated genes, we found an enrichment of terms associated with cell death and apoptosis, supporting that αSyn overexpression led to apoptotic cell death in our cell model, which is in line with the previous observation that caspases were activated upon αSyn overexpression (17). In the GO analysis of the differentially regulated proteome, we found an enrichment of terms associated with vesicular trafficking and synapses, suggesting reorganization of synapses upon αSyn overexpression in LUHMES cells. Two of the synaptic proteins that were downregulated as consequence of αSyn overexpression were syntaxin-binding protein 1, encoded by *STXBP1*, and syntaxin 1B, encoded by *STX1B* with syntaxin-binding protein 1 being one the most strongly downregulated proteins in our cell model and one of the 21 genes/proteins that were differentially regulated on the transcriptomic and proteomic level. Interestingly, Parkin knockout mice, used as an *in vivo* model for PD, also showed dysregulation of *STXBP1* and *STX1B* (31). Mutations in *STXBP1* were associated with mitochondrial dysfunction and young-onset Parkinsonism (32). Moreover, syntaxin-binding protein 1, aka Munc18-1, was shown to act as a chaperone for αSyn with co-expression of Munc18-1, reducing the propensity of mutant αSyn to aggregate. Interestingly, mutant Munc18-1 induced αSyn co-aggregation with Munc18-1 in a cell-free system (33). Moreover, polymorphisms in *STX1B* were previously associated with a higher burden of Lewy bodies in patients with PD (34), and *STX1B* was also identified as a gene being within 250 kB of PD-associated loci in a recent GWAS meta-analysis (9). In light of these data, our data further emphasize a potential role of syntaxin-binding protein 1 (encoded by *STXBP1*) and syntaxin 1B (encoded by *STX1B*) in αSyn pathophysiology, suggesting a direct effect of αSyn overexpression on syntaxin-binding protein 1 (*STXBP1*) and syntaxin 1B (*STX1B*) levels. Another synaptic protein, synaptotagmin 1, encoded by *SYT1* that is involved in ternary SNARE complex formation, was upregulated upon αSyn overexpression. Interestingly, it was previously shown that the function of synaptotagmin 1 is dependent on syntaxin-binding protein 1 (35). It seems possible that the cells induced synaptotagmin 1 translation as a consequence of syntaxin-binding protein 1 (*STXBP1*) downregulation caused by αSyn overexpression. In addition to these, seven more synaptic proteins were downregulated, and 18 more were upregulated as a consequence of αSyn overexpression. Another interesting synaptic protein that was downregulated in our cell model is tyrosine-protein kinase Fyn, encoded by *FYN* (FYN proto-oncogene, Src family tyrosine kinase). *FYN* was also associated with PD in the recent GWAS meta-analysis (9). Furthermore, tyrosine-protein kinase Fyn can directly phosphorylate αSyn at tyrosine 125, and this phosphorylation might be neuroprotective (36).

In addition to proteins that are involved in the vesicular trafficking and synapses, another group of proteins that were regulated in our cell model was associated with the lysosome. One of these was lysosome membrane protein 2, encoded by *SCARB2*. Single nucleotide polymorphisms in *SCABR2* were also previously associated with PD (10, 37, 38). One of the functions of lysosome membrane protein 2 is the transport of the lysosomal ß-glucocerebrosidase (GBA) from the endoplasmic reticulum to lysosomes (39). Notably, mutations in *GBA*, the gene encoding the ß-glucocerebrosidase, are a major genetic risk factor in PD and present in 5–10% of patients (40). Furthermore, it is believed that ß-glucocerebrosidase plays a direct role in degradation of pathological αSyn aggregates (41). It has been shown that lysosome membrane protein 2 expression is critical to β-glucocerebrosidase activity and αSyn clearance (42). Therefore, it seems possible that, upon αSyn overexpression, the cells produce more lysosome membrane protein 2 in order to be able to recruit more glucocerebrosidase to the lysosome, emphasizing the role of both proteins in αSyn pathophysiology. Also, other genes/proteins in the autophagy lysosomal pathway are associated with PD (43, 44). Among these, also cathepsin B, encoded by *CTSB* and sphingomyelin phosphodiesterase, encoded by *SMPD1* (sphingomyelin phosphodiesterase 1), were upregulated in our cell model as a consequence of αSyn overexpression. This further supports that the cells upregulate the lysosomal pathway in order to be able to cope with the increased burden of αSyn. Thus, our findings support the important role of the autophagy-lysosomal pathway in the pathophysiology of PD and related synucleinopathies. Our findings point to a new therapeutic strategy, i.e., modulating lysosome membrane protein 2 expression to increase lysosomal ß-glucocerebrosidase activity to promote αSyn clearance.

There are some shortcomings of our study. The observation that genes that are involved in cell death processes were differentially regulated suggests that the cells were already about to enter the process of dying at the time point chosen for analysis (Day 4 after transduction). Thus, the observations we made might already be an expression of the struggle for survival in the context of an αSyn-induced cell death. However, since LUHMES cells take 6 days after transduction to die, without obvious evidence of cell loss at Day 4 (16), we decided to focus this time point to obtain information about the regulatory processes within the status of deadly stress. Regarding this, LUHMES cells overexpressing αSyn need to be considered as a model that, naturally, cannot reflect all aspects of a disease with a course of decades in human beings. Nevertheless, our data show that our model can still recapitulate many aspects of PD.

In summary, in the transcriptome and proteome analyses in human dopaminergic LUHMES cells overexpressing αSyn, we identified a differential regulation of multiple genes/proteins that had previously been associated with PD. The two most prominent intracellular mechanisms that were differentially regulated were vesicular transport/synapse and the lysosome, both previously associated with the pathophysiology of αSyn and PD. In this respect, our data underline that our cell model recapitulates many aspects of PD pathophysiology and is, therefore, useful to investigate therapeutic approaches to modulate intracellular pathways involved in PD.

## Data Availability Statement

The datasets presented in this study can be found in online repositories. The names of the repository/repositories and accession number(s) can be found at: Sequencing data at NCBI GEO, accession no: GSE191302; Proteomics data at PRIDE, accession no: PXD028322.

## Author Contributions

GH, MH, and KM conceived the study. MH performed the cell culture experiments, prepared the samples for the analysis, performed the overlap, network, and enrichment analysis, created the figures and tables, and wrote the first draft of the manuscript. MS and MK performed transcriptome analysis, mass spectroscopy raw data processing, peptide identification, protein quantification, and proteomics data analysis. MS wrote paragraphs LC–MS/MS Analysis to Proteomics Data Analysis in the Methods section. KP performed the mass spectroscopy and revised paragraph LC–MS/MS Analysis in the Methods section. OC and LD performed the validation experiments. FH helped with interpretation of the data and writing the discussion. ME advised on planning and organization of data analysis. MS, KP, FH, and GH critically revised the manuscript. GH provided overall project leadership. All authors discussed and commented on the manuscript and agreed to publication.

## Funding

Parts of this work have been supported by the German Network for Bioinformatics Infrastructure – de.NBI, service center BioInfra.Prot, funded by the German Federal Ministry of Education and Research (BMBF) – Grant FKZ 031 A 534A. Part of this work was supported by the German Federal Ministry of Education and Research (BMBF) grants de.NBI (Grant No. FKZ 031 A 534A) and i:DSem-Verbundprojekt: Electronic Patient Path (EPP) (Grant No. FKZ031 L 0025A). GH was supported by the German Federal Ministry of Education and Research (BMBF: 01KU1403A EpiPD; 01EK1605A HitTau), Deutsche Forschungsgemeinschaft (DFG, German Research Foundation) under Germany's Excellence Strategy within the framework of the Munich Cluster for Systems Neurology (EXC 2145 SyNergy – ID 390857198), DFG grants (HO2402/6-2, HO2402/18-1 MSAomics), the ParkinsonFonds Germany (hypothesis-free compound screen, alpha-Synuclein fragments in PD), Niedersächsisches Ministerium für Wissenschaft und Kunst (MWK, ZN3440.TP): REBIRTH – Forschungszentrum für translationale regenerative Medizin, Volkswagen Stiftung (NiedersächsischesVorab), and Petermax-Müller Foundation (Etiology and Therapy of Synucleinopathies and Tauopathies).

## Conflict of Interest

The authors declare that the research was conducted in the absence of any commercial or financial relationships that could be construed as a potential conflict of interest.

## Publisher's Note

All claims expressed in this article are solely those of the authors and do not necessarily represent those of their affiliated organizations, or those of the publisher, the editors and the reviewers. Any product that may be evaluated in this article, or claim that may be made by its manufacturer, is not guaranteed or endorsed by the publisher.
