# Supplementary figures and images for: Transcriptome and Proteome Analysis in LUHMES Cells Overexpressing Alpha-Synuclein
Source: Front Neurol. 2022 Apr 11;13:787059. doi: 10.3389/fneur.2022.787059 (PMC9037753; doi:10.3389/fneur.2022.787059)

**A**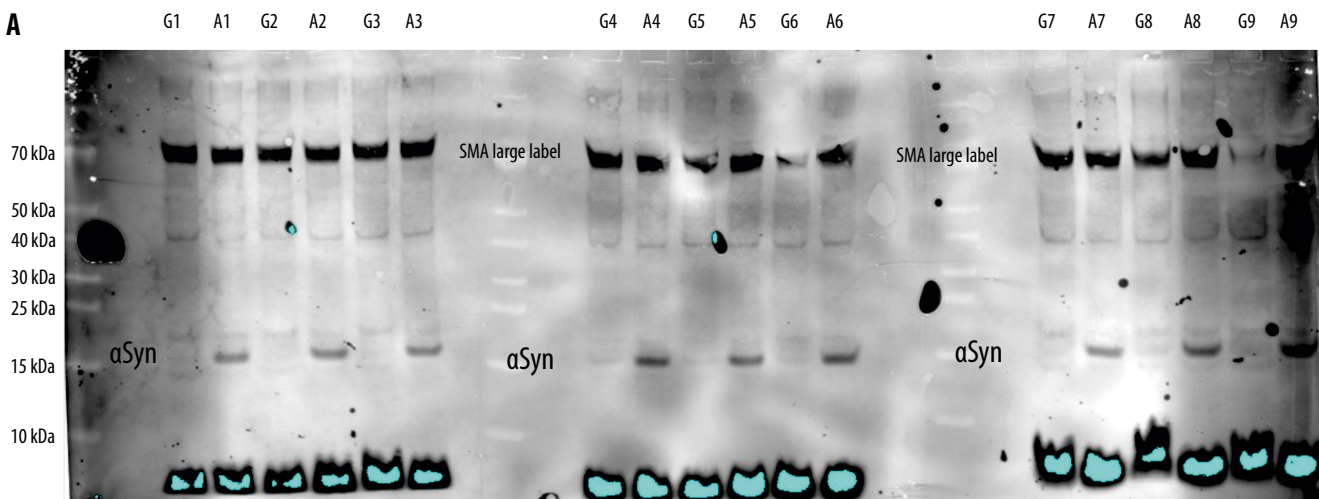**B**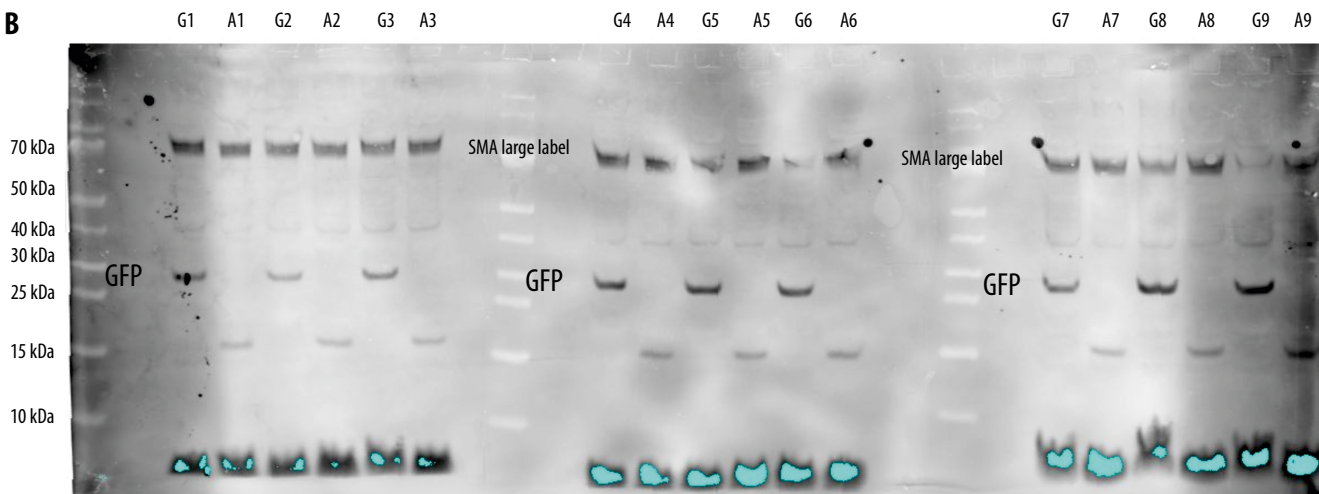

Supplement: Supplementary file 7 [file Data_Sheet_1.PDF]

A

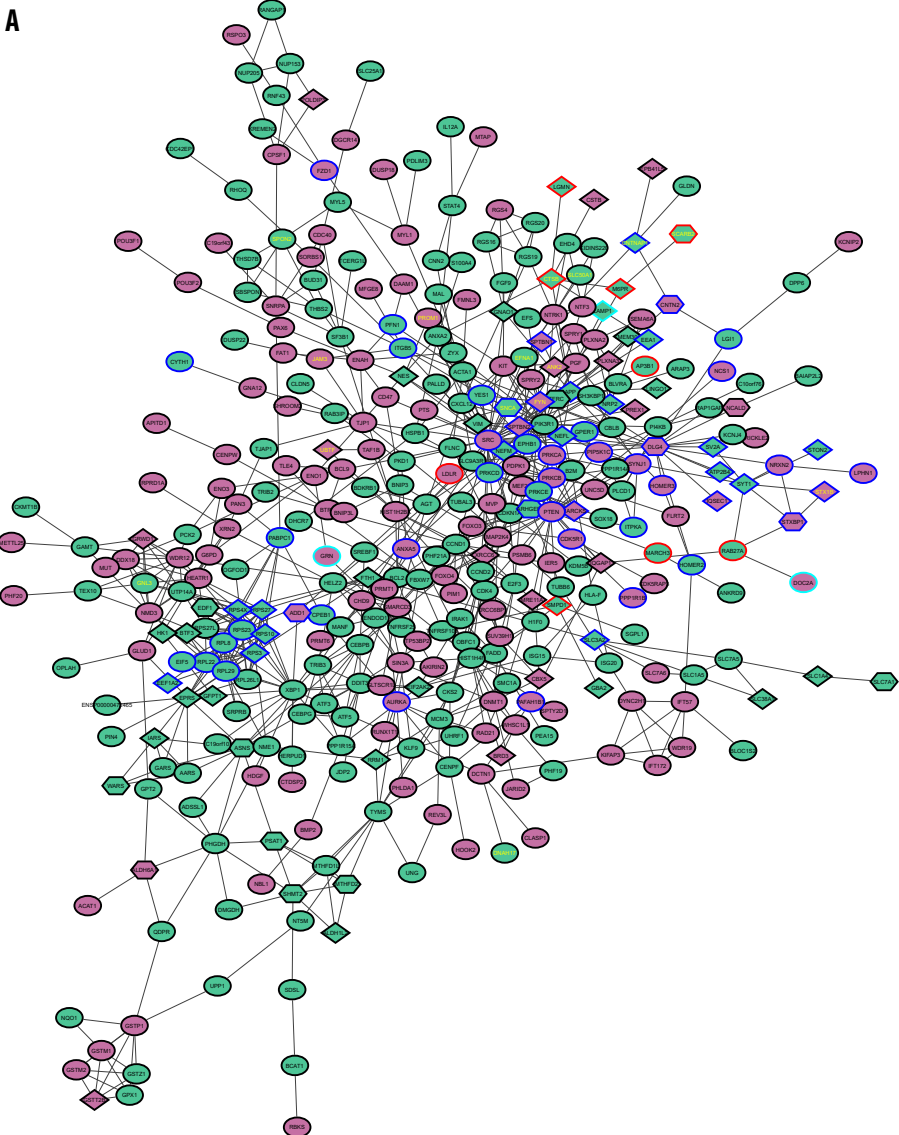

B

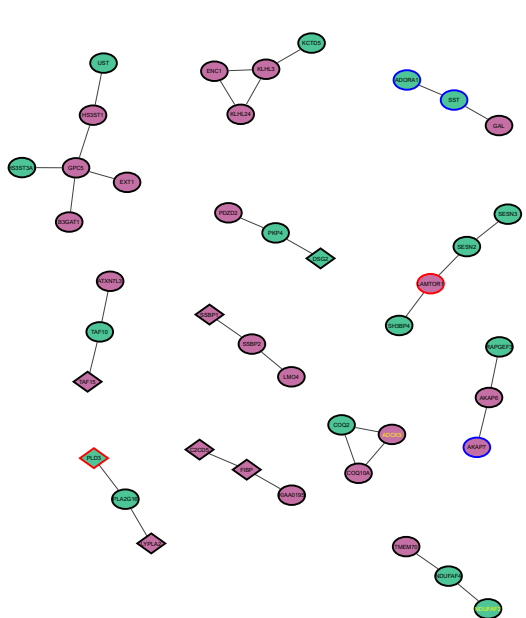

C

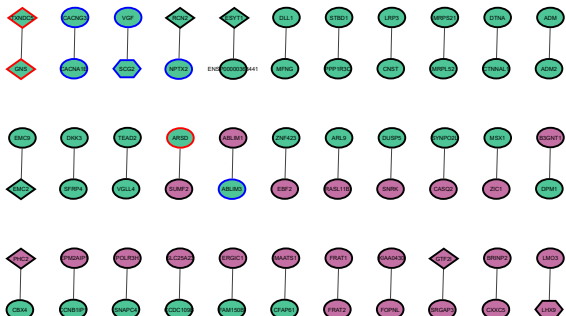

D

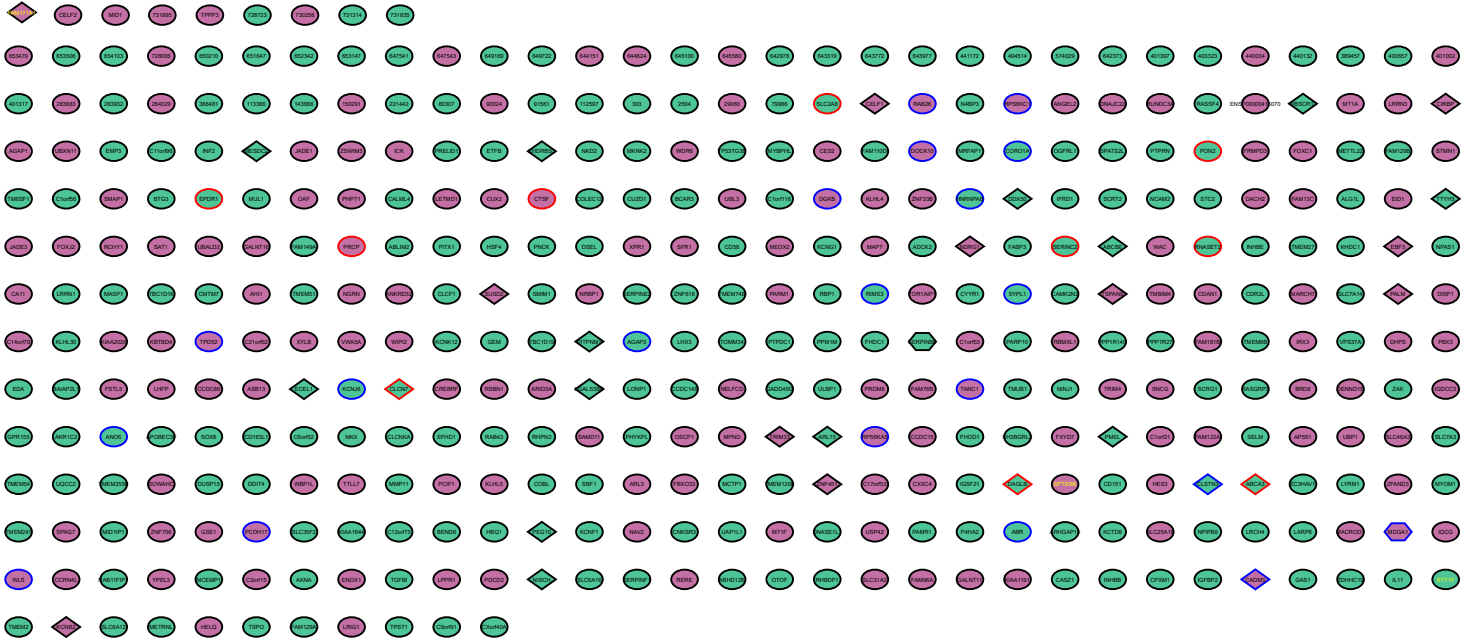

Supplement: Supplementary file 9 [file Data_Sheet_3.PDF]
