# Supplementary material for: Transcriptome and Proteome Analysis in LUHMES Cells Overexpressing Alpha-Synuclein
Source: Front Neurol. 2022 Apr 11;13:787059. doi: 10.3389/fneur.2022.787059 (PMC9037753; doi:10.3389/fneur.2022.787059)
Supplement: Supplementary file 8 [file Data_Sheet_2.PDF]

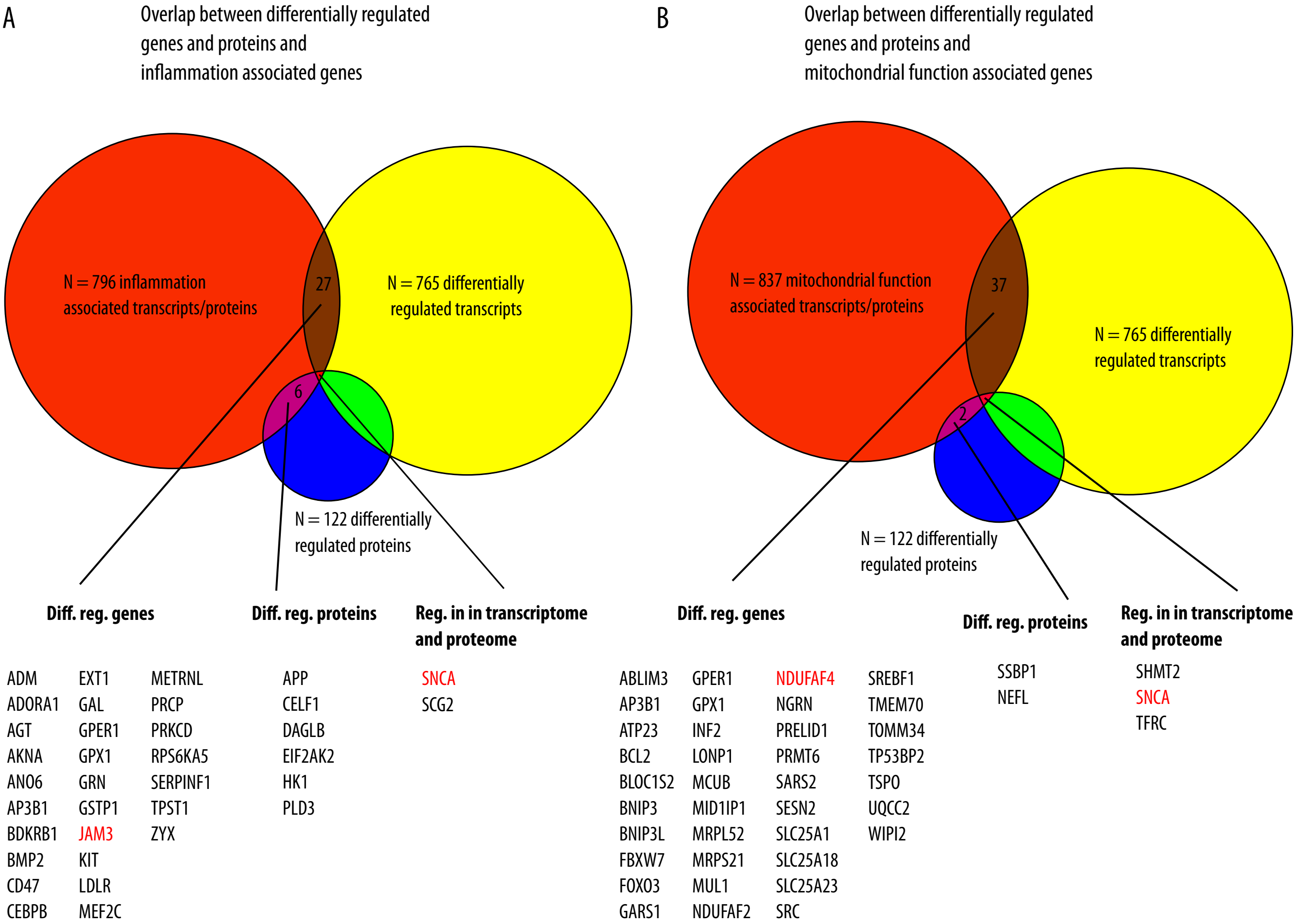
A
Overlap between differentially regulated genes and proteins and inflammation associated genes
N = 796 inflammation associated transcripts/proteins
27
N = 765 differentially regulated transcripts
6
N = 122 differentially regulated proteins
Diff. reg. genes
ADM
ADORA1
AGT
AKNA
ANO6
AP3B1
BDKRB1
BMP2
CD47
CEBPB
EXT1
GAL
GPER1
GPX1
GRN
GSTP1
JAM3
KIT
LDLR
MEF2C
METRNL
PRCP
PRKCD
RPS6KA5
SERPINF1
TPST1
ZYX
Diff. reg. proteins
APP
CELF1
DAGLB
EIF2AK2
HK1
PLD3
Reg. in in transcriptome and proteome
SNCA
SCG2
B
Overlap between differentially regulated genes and proteins and mitochondrial function associated genes
N = 837 mitochondrial function associated transcripts/proteins
37
N = 765 differentially regulated transcripts
2
N = 122 differentially regulated proteins
Diff. reg. genes
ABLIM3
AP3B1
ATP23
BCL2
BLOC1S2
BNIP3
BNIP3L
FBXW7
FOXO3
GARS1
GPER1
GPX1
INF2
LONP1
MCUB
MID1IP1
MRPL52
MRPS21
MUL1
NDUFAF2
NDUFAF4
NGRN
PRELID1
PRMT6
SARS2
SESN2
SLC25A1
SLC25A18
SLC25A23
SRC
SREBF1
TMEM70
TOMM34
TP53BP2
TSPO
UQCC2
WIPI2
Diff. reg. proteins
SSBP1
NEFL
Reg. in in transcriptome and proteome
SHMT2
SNCA
TFRC
