# Supplementary material for: Transcriptome and Proteome Analysis in LUHMES Cells Overexpressing Alpha-Synuclein
Source: Front Neurol. 2022 Apr 11;13:787059. doi: 10.3389/fneur.2022.787059 (PMC9037753; doi:10.3389/fneur.2022.787059)
Supplement: Supplementary file 10 [file Data_Sheet_4.DOCX]

# Supplementary information

**Supplementary methods:**

**Control for successful overexpression of alpha-synuclein or GFP**

The protein samples were divided prior to analysis, in order to perform Western blots as control for successful overexpression of αSyn or GFP. The samples were then dissolved in SPL loading buffer (DyeAGNOSTICS; Halle, Germany) and boiled for 10 min at 95 °C. Thereafter, the samples were loaded on a 12% acrylamide gel. Gel-electrophoresis war performed in 1x MES running buffer. The gel was blotted to a polyvinylidene fluoride (PVDF) membrane. The membrane was then blocked using StartingBlock (Thermo Fisher Scientific, Waltham, MA, United States) for 1h and then incubated with a mouse anti-alpha-synuclein (1:1000; BD Biosciences, Franklin Lakes, NJ, United States) overnight at 4°C. After washing 3-times with TBS for 10 min each time, the membranes were incubated with anti-mouse IRDye800 (1:10.000; LI-COR Biosciences, Lincoln, NE, United States) for 2h at room temperature. Images were taken using the Odyssey Infrared Imaging System 9120 (LI-COR Biosciences).

Thereafter the membrane was incubated again with a mouse-anti-GFP antibody (1:200; Santa Cruz Biotechnology, Dallas, TX, United States), washed three times with TBS for 10 min each time and incubated with an anti-mouse IRDye800 (1:10.000; LI-COR Biosciences) for 2h at room temperature.

**Supplementary figure S1 Confirmation of overexpression of αSyn and GFP prior to proteome analysis**

Western blot performed to confirm successful overexpression of αSyn (**A**) and GFP (**B**). Both panels show the same membrane from a Western blot of all samples used for the proteome analysis. Samples are labeled as G1-9 for those with GFP-overexpression and A1-9 for those with αSyn-overexpression

**A**: The panel shows the membrane after staining with an anti- αSyn antibody. There are strong αSyn bands at ~16 kDa in cells transduced with the adenoviral vector that leads to overexpression of αSyn (A1-9), whereas the samples from the cells that were transduced with the adenoviral vectors that leads to GFP overexpression show only weaker band of endogenous alpha-synuclein (G1-9). The strong bands visible at ~70 kDa is caused by the SMA labelling used as loading control.

**B**: The panel shows the same membrane after staining with an anti-GFP antibody. There are GFP-immunoreactive bands at ~27 kDa in all samples from cells that were transduced with the adenoviral vectors that leads to GFP overexpression (G1-9), whereas there are no bands in samples from αSyn overexpressing cells (A1-9). The bands visible at ~16 kDa caused by a residual signal from the anti- αSyn antibody, the bands at ~70 kDa are caused by a residual signal from the SMA loading control, respectively.

**Supplementary Figure S2 Overlap with genes associated with inflammation and mitochondrial function**

**(A)** Overlaps between N = 796 genes that were present in N = 28 GO terms in the Gene Set Enrichment Analysis (GSEA) database which are associated with inflammation (orange circle) and transcripts (yellow circle) and protein (blue circle) that were differentially regulated in our cell model. Of the 796 genes associated inflammation, N = 29 were differentially regulated in our cell model (brown area). Furthermore, N = 8 proteins encoded by these genes were differentially regulated in our cell model (pink area). In addition to SNCA (alpha-synuclein) that was overexpressed in our cells model, also (SCG2, secretogranin II) was regulated on transcriptomic and proteomic level (red area). (**B)** Overlap between N = 837 genes present that were present in N = 42 GO terms in the Gene Set Enrichment Analysis (GSEA) database which are associated with mitochondria (orange circle) and differentially regulated genes (yellow circle) or proteins (blue circle) in our cell model. N = 40 of the 837 genes associated with mitochondria were differentially regulated in our cell model (brown area). N = 5 corresponding proteins were that were associated with mitochondrial were differentially regulated in our cell model (pink area). Three (SHMT2 [serine hydroxymethyltransferase 2], SNCA [synuclein alpha], and TFRC [transferrin receptor] were differentially regulated in the transcriptome and proteome. Red characters indicate genes/proteins (JAM3, junctional adhesion molecule 3, NDUFAF4, NADH:ubiquinone oxidoreductase complex assembly factor 4) that were found in the recent GWAS meta-analyses.

**Supplementary figure S3 STRING network analysis**

**(A)** Largest interaction network of all differentially regulated genes and proteins with a high confidence level (0.7). **(B)** Smaller interaction networks. **(C)** Genes and proteins with only one interactor **(D)** Genes and proteins with no interactor. Circles indicate items only differentially regulated in the transcriptome. Diamonds show items only differentially regulated in the proteome. Hexagons indicate items that were differentially regulated in both datasets. Upregulation is shown in green, downregulation is shown in purple. Red borders indicate proteins with association to the lysosome. Blue borders indicate proteins with association to synapses. A turquois border indicates association to synapses and the lysosome. A yellow label indicates genes that were previously associated with PD in the recent GWAS meta-analyses.

**Table S1** GO analysis of the whole genome

**Table S2** GO analysis of upregulated genes

**Table S3** GO analysis of downregulated genes

**Tables S4** GO analysis of the whole proteome

**Table S5** GO analysis of upregulated proteins

**Table S6** GO analysis of downregulated proteins
